# Phylogeographic patterning among two codistributed shrimp species (Crustacea: Decapoda: Palaemonidae) reveals high levels of connectivity across biogeographic regions along the South African coast

**DOI:** 10.1371/journal.pone.0173356

**Published:** 2017-03-10

**Authors:** Louisa E. Wood, Sammy De Grave, Savel R. Daniels

**Affiliations:** 1 Department of Botany and Zoology, University of Stellenbosch, Private Bag X1, Matieland, South Africa; 2 Oxford University Museum of Natural History, Parks Road, Oxford, United Kingdom; National Cheng Kung University, TAIWAN

## Abstract

We compare the genetic structuring and demographic history of two sympatric caridean shrimp species with distinct life history traits, one amphidromous species *Palaemon capensis* and one marine/estuarine species *Palaemon peringueyi*, in the historical biogeographical context of South Africa. A total of 103 specimens of *P*. *capensis* collected from 12 localities and 217 specimens of *P*. *peringueyi* collected from 24 localities were sequenced for the mitochondrial cytochrome oxidase one (CO1) locus. Results from analyses of molecular variance (AMOVA), pairwise Φ_ST_ comparisons and haplotype networks demonstrate weak to moderate genetic differentiation in *P*. *capensis* and *P*. *peringueyi* respectively. *P*. *peringueyi* exhibits partial isolation between populations associated with distinct biogeographic regions, likely driven by the region’s oceanography. However, there is minimal evidence for the occurrence of discrete regional evolutionary lineages. This demonstrated lack of genetic differentiation is consistent with a marine, highly dispersive planktonic phase in both the amphidromous *P*. *capensis* and the marine/estuarine *P*. *peringueyi*. Bayesian skyline plots, mismatch expansions and time since expansion indicate that both species maintained stable populations during the Last Glacial Maximum (LGM), unlike other southern African aquatic species.

## Introduction

An understanding of the genetic structuring of organisms is pivotal to a broad range of biological disciplines, including species distribution studies [1], conservation management [2], invasion biology [3], and the elucidation of evolutionary history [4]. In aquatic organisms, population structuring and gene flow patterns are largely determined by the interaction between life history traits such as type of development (direct vs. planktonic), duration of larval period [5], [6], or larval behaviours [7], and historical processes and geographical or environmental barriers acting to either reduce or facilitate gene flow among localities [7]. One specialised life history strategy which has evolved in several families of freshwater decapod crustaceans, gastropod molluscs and teleost fishes inhabiting tropical and sub-tropical habitats is amphidromy [8], [9], [10]; a life history strategy characterised by an adult freshwater phase of growth and reproduction, and a planktonic larval stage that requires a saline component for development [11], [12]. Amphidromy is thought to have evolved in response to the instability of freshwater habitats, with the marine dispersive phase enabling freshwater species to elude drought or cyclonic flood events and recolonise areas following periods of disturbance or water scarcity over long time scales [11]. The characteristic highly dispersive planktonic phase of amphidromous species is an essential factor determining the genetic structuring of populations at both local and regional scales [13]. Marine and amphidromous species often exhibit comparable levels of genetic structuring that is lower than in non-migrating freshwater species and/or marine species with direct development [14]. A number of phylogeographic studies on amphidromous species have revealed a lack of geographic structure and high levels of gene flow over scales from hundreds to thousands of kilometres, e.g. neritid snails [14], [15], gobiid fish [16] as well as atyid and palaemonid shrimps [17], although there are exceptions [18].

**Fig 1:**
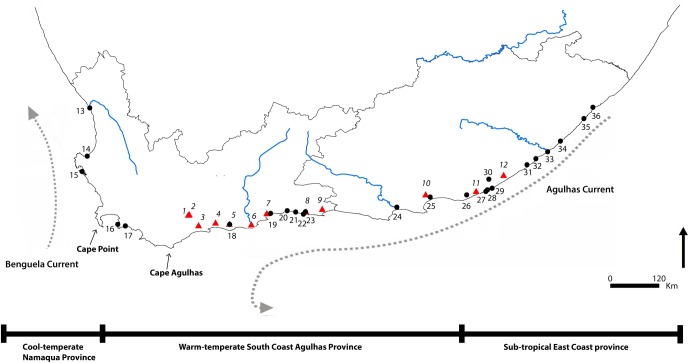
Map of South Africa showing sampling localities for *Palaemon capensis* and *Palaemon peringueyi*. The two major currents, the Benguela and Agulhas currents are shown. *P*. *capensis*: 1, Swellendam; 2, Voorhuis; 3, Malgas; 4, Duiwenhoks; 5, Goukou; 6, Gourits; 7, Little Brak; 8, Knysna; 9, Keurbooms; 10, Sundays; 11, Kowie; 12, Kieskamma. *P*. *peringueyi* 13, Olifants; 14, Berg; 15, Langebaan; 16, Rooiels; 17, Palmiet; 18, Goukou; 19, Great Brak; 20, Touw; 21, Swartvlei; 22, Knysna; 23, Goukamma; 24, Gamtoos; 25, Sundays; 26, Bushmans; 27, Riet; 28, East Kleinmond; 29, Fish; 30, Old Woman’s; 31, Nahoon; 32, Chula; 33, Kei; 34, Qwaninga; 35, Mtata; 36, Umganzana.

Nevertheless, life history strategy alone does not necessarily determine scales of connectivity and population structure, as realized dispersal patterns are also influenced by physical connectivity among coastal and riverine habitats [19]. This in turn may be governed by contemporary barriers to gene flow, such as oceanographic frontal systems [1], [20], upwelling cells [21], environmental characteristics at spawning grounds [22], geographical distances [23], and steep environmental gradients [24], as well as physical historical processes, such as historical climate changes during the Pleistocene which caused fluctuations to sea surface temperatures, sea level, oceanographic circulation patterns and ice sheet coverage [25]. Life-history strategy may affect how species respond to physical barriers [26]. In southern Africa, the population connectivity of marine species is heavily influenced by the region’s oceanic current patterns [2], [27]. The coastline is influenced by two divergent current systems; the cold, high productive Benguela current off the west coast which transports cold water from the poles to the equator, and the warm fast-flowing Agulhas current on the east and south coasts [28]. Along the south-eastern coast of South Africa the currents mix to varying degrees to form discrete biogeographic provinces, with a number of highly dispersive coastal species divided into regionally restricted genetic lineages that are often correlated with these provinces [21], [29], [30], [31], [32], [33], [34], [35]. At least three marine biogeographic provinces are recognised; the cool-temperate Namaqua province on the western coastline (Lüderitz, Namibia to Cape Point, South Africa), the warm-temperate Agulhas province on the southern coastline (Cape Point to Algoa Bay, South Africa) and the sub-tropical East Coast Province on the eastern side (Algoa Bay to the northern borders of the Kwazulu Natal coastline) [36], see Fig 1. The exact spatial boundaries between these provinces are not strictly delineated, as barriers vary across taxa and there are few congruent patterns [2], [32], even with closely related species [37], which forms transition zones. For estuarine organisms an additional barrier to dispersal is presented by South Africa’s highly dynamic estuarine system, whereby approximately 70% of all estuaries are either permanently disconnected from the sea by the formation of sand bars at the mouth (closed estuaries) or are only temporarily open [38]. Under these conditions, it is expected that species with an estuarine phase of their lifecycle could exhibit higher levels of genetic structuring than fully marine species, regardless of larval type or duration, as connectivity is lower [19], [24].

Aside from the interplay between life-history strategy and contemporary barriers to connectivity, long-term historical climatic and oceanographic change are often considered to be drivers of population structuring and demographic changes [1], [22], [27], [30]. In southern Africa, during the last glacial maximum (LGM), approximately 22,000 years ago, the southern coastal plain (Agulhas Bank) became exposed when sea levels dropped to 120m below current levels and water temperatures decreased due to intensified upwelling on the west coast [39], and a reduced influence of the Agulhas Current [40]. How these changes affect the genetic structuring and demographic history of aquatic organisms remains poorly understood, with some marine species maintaining demographically stable populations during the LGM [41], and others showing population bottlenecks and post-LGM colonisation [42]. However, it is hypothesised that the life-history strategy of the species is important in determining its response to climate oscillations [43].

In this study population genetic structuring and past demographic history is explored in species with distinct life history strategies within the context of South Africa’s dynamic biogeographic realm. The caridean shrimp genus *Palaemon* is ideally suited for the exploration of the role of life history strategy on genetic structure and demography, as this genus represents a complex array of life history strategies, from fully marine to fully freshwater with abbreviated larval development, and with a high proportion of amphidromous species [44]. Here we compare a marine/estuarine representative of the genus, *Palaemon peringueyi* (Stebbing, 1915), with the amphidromous species *Palaemon capensis* (De Man *in* Weber, 1897). *P*. *peringueyi* and *P*. *capensis* are suited to comparison since they are sympatric in estuarine areas during the juvenile stage (Wood pers. obs.) where their distribution overlaps and both possess a more saline planktonic larval phase [45], [46]. Nevertheless, they differ in terms of their adult habitat preference, with adult *P*. *peringueyi* inhabiting the marine environment ([46], as *P*. *pacificus*; see [47] for a discussion on the taxonomy of both species) in contrast to the upstream, fully freshwater adult *P*. *capensis* [48]. Occasionally, however, some specimens of the latter species, including ovigerous females, have been recorded from the brackish lower reaches of estuaries [48]. *Palaemon peringueyi* has a broad distribution, from Walvis Bay in Namibia [49] to Kosi Bay along the east coast, which encompasses the three major biogeographic provinces and two transition zones. In contrast, *P*. *capensis* is restricted to an area bounded by the Palmiet River on the south coast and the Kieskamma River on the east coast [48], which comprises the warm-temperate Agulhas province and south-east transition zone. Although no previous genetic work has been carried out on *P*. *capensis*, an earlier study [50], indicated that *P*. *peringueyi* exhibited significant genetic structuring across its distribution, with distinct evolutionary lineages correlated with the three biogeographic provinces. Here we further this work by sampling more intensely across and within putative biogeographic regions, with a focus on collecting from both open and closed estuary systems. This study thus has two major aims: 1) to compare the level of population genetic structuring in a marine and amphidromous species of *Palaemon* according to biogeographic region, 2) to determine whether each of the species shows similar patterns of demographic history. It is hypothesised that patterns of population sub-structuring in both species will coincide with known biogeographic breaks in species distributions. Specifically, populations of *P*. *capensis* will be genetically dissimilar across the south-east transition zone, and in *P*. *peringueyi* populations from the cool-temperate Namaqua province, warm-temperate Agulhas province and sub-tropical East Coast Province will be genetically distinct. The data presented here presents evidence that both species exist as single evolutionary lineages across their South African distribution range, contrary to the findings in [50], and show similar patterns of demographic stability during the LGM.

## Materials and methods

### Shrimp collection and sampling

Between 7–12 specimens of *Palaemon capensis* (n = 103) and *P*. *peringueyi* (n = 217) were collected from 12 and 24 localities respectively along the South African coastline between 2015 and 2016, encompassing the entire distribution of each species in South Africa (Fig 1, Table 1). Specimens were collected using handnets and anaesthetised using clove oil and preserved in the field in 95% ethanol immediately after collection. Species were identified using [47], voucher specimens were deposited in the collections of the South African Museum (SAM C, IZIKO Museums of Cape Town South Africa), under accession numbers SAMC-A088831–40. Sampling was carried out under permits from CapeNature and SanParks.

**Table 1:** Sampling localities for the two shrimp species throughout their distribution in South Africa with the number of specimens collected with genetic diversity indices at each locality based on mtDNA sequences.

Locality	Map reference	*N*	Coordinates	*Nh*	*Nph*	*Np*	*h*	*π*_*n*_	*A*_r_
***Palaemon capensis***									
South Coast									
Malgas	1	9	S 34°18.274' E 20°37.047'	4	0 (0%)	3	0.810	0.0024	
Swellendam	2	7	S 34°04.174' E 20°24.898'	6	3 (50%)	6	0.833	0.0038	
Voorhuis	3	9	S 34°04.170' E 20°23.378'	4	1 (25%)	5	0.583	0.0033	
Duiwenhoks	4	10	S 34°15.089' E 20°59.501'	7	2 (29%)	6	0.867	0.0038	
Goukou	5	10	S 34°16.279' E 21°18.020'	6	2 (33%)	5	0.844	0.0034	
Gourits	6	8	S 34°17.201' E 21°47.555'	4	2 (50%)	4	0.643	0.0025	
Little Brak	7	10	S 34°02.377' E 22°07.953'	7	3 (43%)	7	0.933	0.0042	
Knysna	8	1	S 33°59.894' E 23°00.181'	1	0 (0%)	0	1.000	0.0000	
Keurbooms	9	10	S 33°56.326' E 23°21.967'	2	1 (50%)	3	0.250	0.0016	
**Pooled South Coast**		**74**		**21**	**14 (66%)**	**21**	**0.748**	**0.0027**	**N/A**
South-East Coast									
Sundays	10	10	S 33°36.911' E 25°40.008'	1	0 (0%)	0	0.000	0.0000	
Kowie	11	10	S 33°32.753' E 26°47.184'	2	1 (50%)	1	0.200	0.0004	
Kieskamma	12	9	S 33°11'057' E 27°23'257'	1	0 (0%)	0	0.000	0.0000	
**Pooled South-East Coast**		**29**		**2**	**1 (50%)**	**1**	**0.069**	**0.0001**	**N/A**
**Overall *P. capensis***		**103**		**22**	**15 (68%)**	**22**	**0.607**	**0.0024**	**N/A**
***Palaemon peringueyi***									
West Coast									
Olifants	13	10	S 31°42'136’ E 18°11'574’	5	1 (20%)	5	0.844	0.0026	
Berg	14	12	S 32°47.213’ E 18°08.627’	4	0 (0%)	4	0.742	0.0023	
Langebaan	15	10	S 33°07.082’ E 18°00.467’	2	0 (0%)	1	0.356	0.0006	
**Pooled West Coast**		**32**		**5**	**1 (20%)**	**5**	**0.685**	**0.0019**	**3.194**
South-West Coast									
Rooiels	16	10	S 34°17.968' E 18°49.415'	4	0 (0%)	3	0.822	0.0020	
Palmiet	17	10	S 34°20.396' E 18°59.508'	4	0 (0%)	3	0.800	0.0022	
South-Coast									
Goukou	18	8	S 34°17.939' E 21°18.826'	3	0 (0%)	2	0.714	0.0017	
Great Brak	19	10	S 34°03.135' E 22°13.157'	5	1 (20%)	3	0.800	0.0020	
Touw	20	9	S 33°59.549' E 22°35.276'	4	0 (0%)	3	0.750	0.0019	
Swartvlei	21	10	S 34°01.354' E 22°46.552'	5	1 (20%)	4	0.756	0.0025	
Knysna	22	10	S 34°01.284' E 22°59.540'	5	0 (0%)	3	0.844	0.0022	
Goukamma	23	8	S 34°04.001' E 22°56.702'	4	0 (0%)	4	0.786	0.0030	
Gamtoos	24	10	S 33°54.985' E 25°01.591'	4	0 (0%)	3	0.778	0.0019	
South-East Coast									
Sundays	25	9	S 33°41.185' E 25°46.394'	4	0 (0%)	3	0.750	0.0019	
Bushmans	26	10	S 33°38.497' E 26°34.519'	2	0 (0%)	1	0.533	0.0009	
Riet	27	7	S 133°33.280’ E 27°00.380’	5	1 (20%)	7	0.857	0.0040	
East Kleinmond	28	7	S 33°32.042' E 27°02.510'	4	0 (0%)	3	0.857	0.0021	
Fish	29	9	S 33°29.200’ E 27°07.390’	6	1 (16%)	5	0.889	0.0028	
Old Woman’s	30	10	S 33°28.539’ E 27°08.425’	3	0 (0%)	2	0.511	0.0010	
Nahoon	31	9	S 32°57.972' E 27°55.147'	5	0 (0%)	4	0.861	0.0024	
Chula	32	7	S 32°50.141’ E 28°06.760’	3	0 (0%)	5	0.667	0.0033	
Kei	33	10	S 32°40.587' E 28°22.774'	5	0 (0%)	3	0.822	0.0021	
Qwaninga	34	7	S 32°26.283' E 28°40.039'	4	1 (25%)	5	0.857	0.0041	
**Pooled South Coast**		**170**		**12**	**5 (42%)**	**10**	**0.793**	**0.0024**	**4.451**
East-Coast									
Mtata	35	7	S 31°56.632' E 29°11.038'	2	0 (0%)	1	0.476	0.0008	
Umganzana	36	8	S 31°41.373' E 29°22.994'	4	2 (50%)	4	0.750	0.0025	
**Pooled East Coast**		**15**		**4**	**2 (50%)**	**4**	**0.600**	**0.0017**	**3.000**
**Overall *P. peringueyi***		**217**		**16**	**8 (50%)**	**13**	**0.795**	**0.0025**	**N/A**

*N* number of samples, *Nh* haplotype number, *Nph* number of private haplotypes, *Np* number of polymorphic sites, *h* haplotype diversity, *π*_*n*_ nucleotide diversity, *A*_r_ allelic richness after rarefaction to 15 individuals.

### DNA extraction and sequence alignment

DNA was extracted from abdominal muscle or pleopods using the NucleoSpin® Tissue extraction kit (Machery-Nagel, Germany) following the manufacturers’ protocol. Extracted DNA was stored at -20°C until required for polymerase chain reaction (PCR). The mitochondrial CO1 gene was selected for the current analysis as it is widely used in studies on palaemonid shrimp [51], and is highly variable, facilitating comparison with previous work.

For *P*. *peringueyi* the CO1 region amplified with the primer pair LCO11490 and HCO2198 [52], however for *P*. *capensis* two species specific internal primers were designed for successful amplification: *Shrimp F* (5’–CGTCACAGCCCATGCATTC -3’) and *Shrimp R* (5’–TAGAGAATCGGGTCTCCTCCT -3’). This latter set of primers amplified an approximately 500-bp fragment of CO1. See S1 Table for the list of primer pair sequences and the PCR profile.

The PCR products were electrophoresed for four hours in a 1% agarose gel and subsequently purified using the BioFlux purification kit (Bioer Technology Co., Ltd). Purified products were cycle-sequenced using BigDye (Applied Biosystems [ABI]) and analyzed on a 3730 automated sequencer (ABI).

### Data analysis

Sequences were visualised using BioEdit v7.1.3.0 [53], and checked for ambiguities. Alignments were performed in BioEdit’s ClustalW Multiple Alignment tool [54]. To check for stop codons, CO1 sequences were translated to amino acids using the online programme EMBOSS-Transeq (http://www.ebi.ac.uk/emboss/transeq/). No stop codons were detected.

### Population genetic analysis

The software ARLEQUIN version 3.5.1.2 [55], was used to analyse population genetic structure of each species. The standard diversity indices calculated for each sampling location and across each biogeographic region were number of haplotypes (*H)*, haplotypic diversity (*h*), number of private haplotypes (*Nph*), number of polymorphic sites (*Np*), and nucleotide diversity (*π*_*n*_). In addition allelic richness (haplotype richness) was estimated by rarefaction using CONTRIB v 1.02 for each biogeographic region, where possible [56]. The rarefaction method was used to standardise the allelic richness across biogeographic regions by correcting variation in sample sizes, ensuring that the rarefaction size was not larger than the smallest sample size [56].

Estimation of genetic differentiation within and among sample localities was conducted using different approaches. Firstly pairwise Φ_ST_ values were estimated between all sampled localities in ARLEQUIN, with significance at the 0.05 level determined by 10,000 permutations. Secondly, evidence of population genetic differentiation within each CO1 dataset was assessed using a one-level global analysis of molecular variance (AMOVA) in ARLEQUIN. The programme jMODELTEST 2 [57], was used to obtain the best-fit substitution model for each species using the Akaike information criterion (AIC) [58], with the HKY + *I* model selected for *P*. *capensis* and the TIM2 + G model selected for *P*. *peringueyi*. However, as neither model is implemented in ARLEQUIN, distances were estimated using the most similar substitution model, Tamura–Nei. In addition, hierarchical analysis of molecular variance was carried out to evaluate population sub-structuring hypotheses of differentiation between biogeographic regions by identifying groups of sites that maximised the statistic Φ_CT._ AMOVA tests were conducted to investigate: i) the influence of the south-east coast transition zone (e.g. Algoa Bay) on the genetic structuring of *P*. *capensis*; ii) the separation between the sub-tropical east coast population, warm-temperate south coast population and cool-temperate west coast population of *P*. *peringueyi*; iii) the separation between the sub-tropical east coast population and the warm-temperate south/ cool-temperate west coast population of *P*. *peringueyi*. In all analyses 10,000 permutations were specified to test for significance. In order to test for possible isolation by distance (IBD), pairwise Φ_ST_ values among localities [59], and corresponding pairwise geographic distances were tested by performing Mantel tests in XLSTAT v.5.1 software (http://www.xlstat.com: Addinsoft, New York) using 10000 permutations. Geographic distances between localities were determined using GoogleEarth as the shortest along-coast distance between sites but excluding bays.

To depict the evolutionary relationships among the mtDNA haplotypes, a haplotype network was constructed using TCS 1.21 set at 95% confidence [60]. The shortest tree was chosen on the basis of a coalescent theory approach, whereby haplotypes are linked to the most abundant and/or geographically closest occurring haplotype [61].

### Demographic history

To further investigate the underlying demography of both species Tajima’s D [62], and Fu’s F [63], statistics were first calculated in ARLEQUIN to test for deviations from neutrality with significance determined using 10,000 permutations. Pairwise mismatch distributions [64], were calculated in ARLEQUIN to test for population expansion using 10,000 permutations. The Sum of Squared deviations and Harpening’s Raggedness Index were calculated to test for the goodness of fit of the data in the mismatch analysis. If significant population growth was detected, the time of expansion was calculated with the formula T = τ/2 μ, where μ = generation time × number of base pairs per sequence × mutation rate for the marker used, and τ was calculated in ARLEQUIN. A mutation rate of 1.4% per million years [65], and a generation time of 1.5 years were used for the calculations. This generation time was used as the average of that reported for other Palaemonidae [66], [67]. To provide a temporal perspective on demographic events, Bayesian Skyline Plots [68], were constructed in BEAST 1.7.5 [69], and Tracer 1.5 for each identified genetic cluster [70]. Analyses were run using a strict molecular clock, implementing a mutation rate of 1.4% per million years [65], and assuming an HKY + *I* or TIM2 + G model of evolution for *P*. *capensis* and *P*. *peringueyi* respectively. Two independent runs were performed for 100 million MCMC generations, sampling every 1,000 generations. Runs were evaluated in Tracer 1.5 [70] to assess convergence and visualisation of median and 95% highest posterior probability density intervals (HPD), using the effective sample size (ESS > 200) as an indicator.

## Results

In total 103 specimens of *Palaemon capensis* and 217 specimens of *Palaemon peringueyi* were sequenced for the CO1 locus. Sequences were deposited in GenBank (accession numbers KY660277 –KY660314). After editing and alignment in CLUSTAL X, 477 bp and 578 bp of the CO1 fragment were used in the analysis for *P*. *capensis* and *P*. *peringueyi* respectively. For *P*. *capensis* there were 22 polymorphic sites, seven of which were parsimony informative, whilst for *P*. *peringueyi* there were a total of 13 polymorphic sites, eight of which were parsimony informative.

### Population genetic analysis

**Fig 2:**
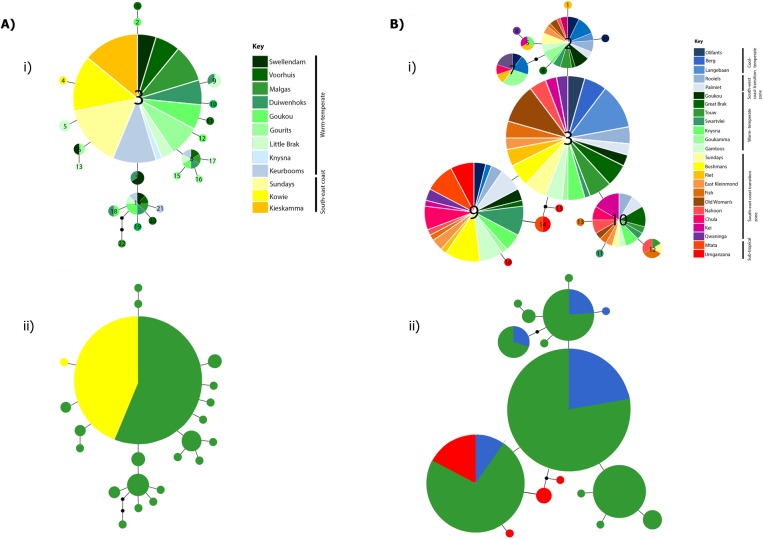
**A) Haplotype networks for *Palaemon capensis*.** Size of the circles are representative of the number of individuals with that haplotype. The smallest circles represent a haplotype frequency of one. Each connecting line represents one mutation step between haplotypes and black circles are representative of an additional mutational change. In i) the numbers within circles represent the haplotype number and correspond to S2 Table; ii) the colours represent biogeographic region and correspond to the south coast populations (green) and south-east coast populations (yellow). **B) Haplotype network for *Palaemon peringueyi*.** Size of the circles are representative of the number of individuals with that haplotype. The smallest circles represent a haplotype frequency of one. Each connecting line represents one mutation step between haplotypes and black circles are representative of an additional mutational change. In i) the numbers within circles represent the haplotype number and correspond to S3 Table; ii) the colours correspond to biogeographic region and represent east-coast populations (red); south-coast populations (green) and west-coast populations (blue).

The parsimony haplotype networks constructed recovered no clear phylogeographic structure for either *species* (Fig 2A and 2B). Within *P*. *capensis* haplotype diversity was lower (0.607) than within *P*. *peringueyi* (0.795) with 22 haplotypes (seven shared and 15 unique) and 16 haplotypes (eight shared and eight unique) detected respectively. The nucleotide diversity of both *P*. *capensis* and *P*. *peringueyi* was low at 0.00237 and 0.00247 (Table 1), reflecting the fact that in both species most individuals shared a low number of common haplotypes. In *P*. *capensis* one dominant haplotype (H3) occurred in 62% of individuals whilst in *P*. *peringueyi* three dominant haplotypes (H2, H3, H9) were present in 19%, 31% and 24% of individuals respectively.

Within *P*. *capensis*, mtDNA genetic diversity was higher on the south coast than on the south-east coast, with the highest haplotype diversity i.e. highest percentage of unique haplotypes also found in south coast populations (Table 1).

Within *P*. *peringueyi*, mtDNA genetic diversity was generally highest on the south and south-east coast, with lower values recorded on the east and west coasts (Table 1). Broadly speaking haplotype diversity was highest on the south and east coast, with the lowest number of private haplotypes being found on the west coast (Table 1). Allelic richness was higher for the southern biogeographic region, than for the eastern and western biogeographic region.

For *P*. *capensis* the one-level overall AMOVA results suggests shallow, but significant genetic differentiation among the 12 sampled localities (Φ_ST_ = 0.02, *p* < 0.05) (Table 2). Pairwise Φ_ST_ values were mostly not significant, except in the case of localities at the periphery of the distribution, i.e. on the extreme west coast locations (Swellendam/Voorhuis) versus east coast locations (Sundays/Kowie/Kieskamma) (see S4 Table). When sampling localities were partitioned into south coast and south-west populations in accordance with the positioning of the proposed south-east transition zone, AMOVA results indicated low but significant differentiation among groups (Φ_CT_ = 0.096, *p* < 0.05), suggesting that the hypothesis that gene flow restrictions occur around the south-west transition zone i.e. Algoa Bay cannot be rejected. However this apparent population genetic structure could be caused by the significant correlation between linearised genetic and geographic distances (i.e. Mantel test *r =* 0.391, *p* < 0.05).

**Table 2:** Analysis of molecular variance (AMOVA) results based on COI data, for the five population differentiation scenarios tested.

	One-level *P*. *capensis*	One-level *P*. *peringueyi*	Warm-temperate vs south-east transition zone *P*. *capensis*	Sub-tropical east vs. warm-temperate/cold *P*. *peringueyi*	Sub-tropical east vs warm-temperate vs cold *P*. *peringueyi*
**Among groups % variation**	2.77	13.35	9.61	34.22	18.29
**Within groups % variation**	N/A	N/A	-1.70	4.80	4.95
**Within populations % variation**	97.23	86.65	92.09	60.99	76.76
**Fixation index**	Φ_ST_ = **0.028**	Φ_ST_ = **0.133**	Φ_CT_ = **0.096**	Φ_CT_ = **0.342**	Φ_CT_ = **0.183**

Statistically significant results (p<0.05) in bold.

The one-level AMOVA results for *P*. *peringueyi* indicated a mean overall Φ_ST_ = 0.133 (*p* < 0.05) (Table 2). Pairwise Ф_ST_ analysis showed that significant Φ_ST_ values were between the sub-tropical east coast and all other sample localities and ranged from 0.16 < Φ_ST_ < 0.73, suggesting the presence of a gene flow barrier between Qwaninga and Mtata (S5 Table). Less pronounced, but significant structure can also be observed within the Goukamma (0.21 < Φ_ST_ < 0.57) and Bushmans (0.19 < Φ_ST_ < 0.47) river basins. Hierarchical analysis of the distribution of genetic variance corroborated the pairwise Ф_ST_ tests, in finding that an eastern versus west/southern subsystem population structure was the best explanation of the distribution of genetic variance (Φ_CT_ = 0.34, *p* < 0.05). The Mantel test indicated a significant relationship between linear genetic and geographic distances for *P*. *peringueyi* (*r* = 0.163, *p* < 0.05).

### Demographic history

**Fig 3:**
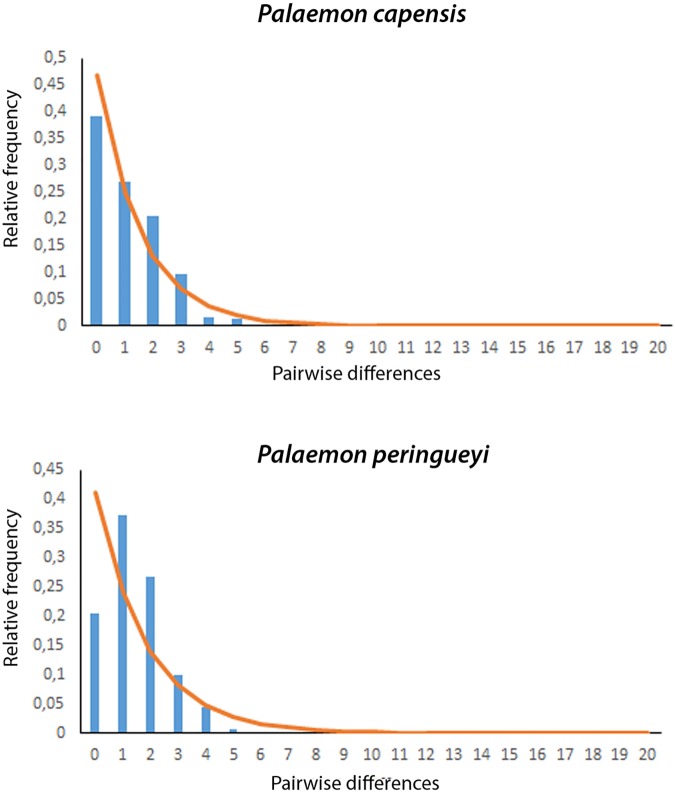
Mismatch distributions for *P*. *capensis* and *P*. *peringueyi* with the line showing model distribution under the sudden population expansion model; x axis = pairwise differences and y axis = frequency.

Due to the shallow population structure indicated by AMOVA for *P*. *capensis* and the limited sample size obtained for the eastern sub-population (n = 15) for *P*. *peringueyi*, demographic analyses were performed only on the pooled datasets across all locations for both species. The CO1 data for *P*. *capensis* revealed significant departures from neutrality expectation with Tajima’s D and Fu’s Fs for the complete dataset (Tajima’s D -2.16, *p* < 0.01; Fu’s F -22.06, *p* < 0.01). A pattern of population expansion for *P*. *capensis* was supported by the unimodal mismatch analysis, as well as by the sum of squared deviations (SSD) and the Raggedness Index which were not statistically different at the 95% confidence level from model predicted frequency (SSD 0.0015, *p* > 0.05; Raggedness Index 0.0447, *p* > 0.05) (Fig 3). These results are consistent with a recent demographic expansion after a bottleneck or selective sweep event. The value of τ for the entire dataset was 2.0234, corresponding to an initiation of demographic expansion of approximately 101,000 years before present for *P*. *capensis* in South Africa.

For *P*. *peringueyi* Fu’s F for all localities combined was negative and significant (Fu’s F -6.29, *p <* 0.05), but Tajima’s D was non-significant (Tajima’s D -0.849, p *>* 0.05). The combined dataset had a unimodal distribution, and the SSD value did not differ statistically from that expected for populations experiencing a population expansion (SSD 0.0028, *p* > 0.05) (Fig 3). The value of τ for the entire dataset was 1.469, corresponding to an initiation of demographic expansion of approximately 60,512 years before present for *P*. *peringueyi* in South Africa.

**Fig 4:**
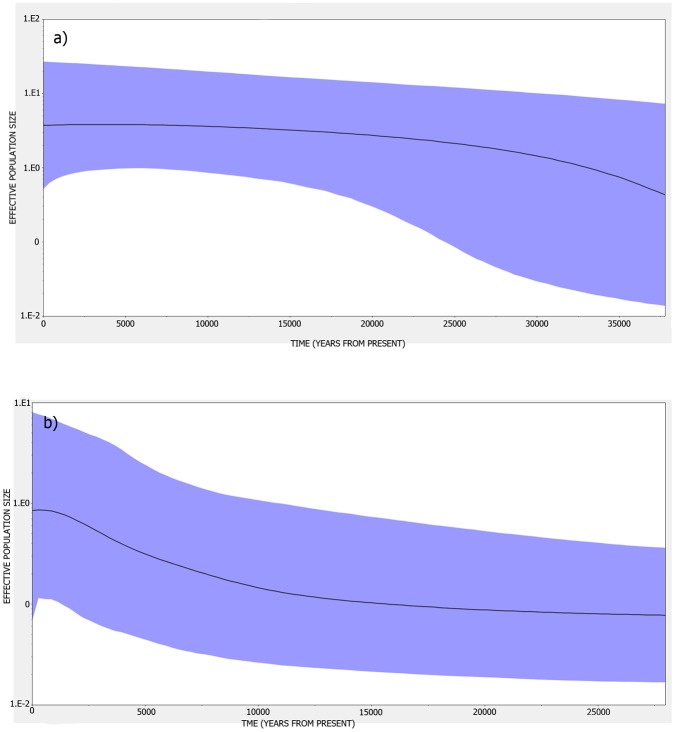
Bayesian Skyline Plots (BSPs) for a) *P*. *capensis* and b) *P*. *peringueyi* across their South African distribution range. The solid line indicates the median estimate, and the 95% HPD interval is depicted in blue.

Bayesian Skyline Plot analyses support mismatch analyses in indicating that both *P*. *capensis* and *P*. *peringueyi* populations remained stable during the last glacial maximum approximately 18,000 years ago (Fig 4). In both species the slope of the line indicates a gradual expansion, although the effective population size has remained fairly constant, at least for the last 25,000 (*P*. *peringueyi*) to 35,000 (*P*. *capensis*) years.

## Discussion

### Population structure and phylogeographic study

Understanding the population structure of a species in relation to its distribution can help in the identification of biogeographical barriers and filters to gene flow or ‘genetic breaks’ and provide key insights into the evolutionary history and life history strategy of the species. In contrast to previous work on one of the species [50], our results present evidence that both *P*. *capensis* and *P*. *peringueyi* exist as single evolutionary lineages throughout their South African range.

Nevertheless, analysis of within population genetic differentiation does suggest that there is a limited degree of population sub-structuring in *P*. *capensis* and *P*. *peringueyi*. The observed population structure in both shrimp species is intermediate in degree between two extremes previously reported for the South African biogeographic region, which range from species occurring in a single, highly connected, panmictic population, e.g. the goby *Psammogobius knysnaensis* (*see* [71]), to others with strong genetic differentiation over short distances, e.g. the mudprawn *Upogebia africana* (*see* [29]), the sciaenid fishes *Atractoscion aequidens* (*see* [21]) and *Argyrosomus inodorus* (*see* [33]), as well as the clinid fish *Clinus cottoiides* (*see* [31]) and the mussels *Perna perna* and *Mytilus galloprovincialis* (*see* [30]).

For the amphidromous prawn, *P*. *capensis*, the low level of population differentiation presented by the AMOVA results indicates wide ranging marine dispersal and high connectivity across sampling localities. As such, none of the oceanographic features which characterise the southern to south-western coastline, such as the fragmentation of freshwater habitat, the dynamic nature of estuarine systems (i.e. open and closed) nor oceanic currents have created significant barriers or filters to gene flow. This shallow population structuring likely reflects the biological characteristics of *P*. *capensis*. Although the maximal larval dispersal range of *P*. *capensis* remains unknown, on the basis of the present results it can be postulated to be relatively high, as the life cycle of this species is characterized by non-abbreviated planktonic larval stages [49], typical of the majority of coastal and amphidromous Palaemonidae [72]. The duration of the life-cycle is unknown, but is very likely to be in the range delineated by 5–16 days in freshwater restricted *Palaemon* species [73], [74], to 29 days in *P*. *peringueyi* [46]. Analysing samples of *P*. *capensis* across its entire 700 km distribution range showed that genetic structure was only observed at the extremities of the range. Pairwise Ф_ST_ comparisons indicated significant genetic differentiation between Swellendam and Voorhuis on the south coast versus Kowie, Sundays and Kieskamma on the east coast, with isolation by distance confirmed by the Mantel test. This result correlates with the general notion that genetic differentiation of populations of amphidromous shrimp is rare or at most weakly expressed over larger scales (> 1000 km) [75]. A similar result was encountered in the distantly related palaemonid species, *Cryphiops caementarius* (see [76]), as well as in amphidromous representatives of the Atyidae [17], the other main family of freshwater shrimps. Interestingly, the hierarchical AMOVA suggests that genetic differentiation does fall within a widely discussed biogeographic barrier between the southern populations and the south-east transition zone in the proximity of Algoa Bay [27]. Here, both oceanographic dispersal limits as well as increased genetic drift within a peripheral population may cause genetic structuring at the limits of *P*. *capensis* geographic range.

Contrary to predictions, the broadly distributed estuarine/marine shrimp species *P*. *peringueyi* shows only weak genetic structuring across its range, somewhat coinciding with known biogeographic boundaries. Whilst the shared haplotypes found for the entire CO1 dataset indicate that this shrimp species can disperse over long distances, suggestive of long planktonic larval duration, *P*. *peringueyi* does not form a true panmitic population as several sampled localities are differentiated. Pairwise Ф_ST_ analysis indicated that populations from the sub-tropical east coast of South Africa were genetically dissimilar to populations from the cold west/ temperate south coast with the location of the genetic break occurring between Qwaninga and Mtata. This finding is concordant with previous studies on marine invertebrates which found similarly located phylogeographic breaks at the boundary between the warm-temperate Agulhas province and the sub-tropical east coast province [30], [35]. The exact location of the phylogeographic break is typically species-specific, with the northernmost breaks having been identified on the Central Wild Coast (Transkei region) [29], and the southernmost breaks near Algoa Bay [31]. Genetic discontinuity at the warm-temperate to sub-tropical transition zone has been attributed to the offshore deflection of the Agulhas current, which potentially carries entrained larvae away from the intertidal zone [27] or by selection pressure created by a sudden drop in temperature in this region [77]. The significant genetic differentiation of Mtata and Umganzana may suggest that on a finer geographic scale, dispersal of *P*. *peringueyi* is limited by regional currents patterns or by the local retention of larvae in the water column. Alternatively, genetic differentiation may reflect thermal adaptation of larvae across adjacent, temperature-defined biogeographic regions [77].

It is evident from the present study that *P*. *peringueyi* does not show the deeply divergent lineages that were resolved in [50]. Estimates of genetic divergence between the CO1 haplotypes in [50], and those of the current study indicated that whilst two of the 12 haplotypes in [50], fell within species limits at 0.3–1.1% divergence, for ten haplotypes divergence ranged from 5.2% to 11%. This was supported by the topologies obtained by a further maximum likelihood analysis (see S1 Fig) in which the 12 CO1 haplotype sequences generated in [50], on GenBank were added to the sequences generated in the current dataset. Although *P*. *peringueyi* formed a monophyletic clade, at least three clades were observed within a broader *P*. *peringueyi* taxon with the inclusion of the additional sequences. All *P*. *peringueyi* sequences generated in this study, sampled from localities across the three biogeographical provinces, formed a well-supported clade with two haplotypes generated in [50], from the cold western lineage only, with the remaining 10 haplotypes (from [50]) forming two separate groupings. The mean CO1 genetic divergence between clades was comparable to that reported for cryptic species in related species of Palaemonidae (e.g. *Macrobrachium potiuna* [78]). However, given the intensive sampling effort of the present study with 217 specimens collected herein compared to 42 specimens in [50], and the complete spatial overlap between the sample localities in both studies, it would be surprising if such cryptic variation, if indeed present, would not be recovered in the current dataset. It is possible, although very unlikely given the general conservative ecology of *Palaemon* species, that these supposed cryptic species exhibit seasonal variation in their abundance or habitat preference and lifestyle, and thus were simply not sampled herein, as sampling was largely (but not exclusively) restricted to summer months with the majority of samples obtained from either *Zostera capensis* beds in estuaries, or reed beds in the lower reaches of rivers.

### Demographic history

For both shrimp species the combination of high haplotypic diversity and low nucleotide diversity is typical of a population that has undergone relatively recent population expansion after a bottleneck or founder event and is often associated with negligible population structure. Similarly, the typical ‘star-like’ haplotype network for *P*. *capensis* and the more complex ‘star-like’ type haplotype network for *P*. *peringueyi*, showing several common haplotypes are indicative of growing populations. Based on mismatch distribution estimates and BSP plots the timing of the expansion pre-dates the LGM and is indicative of an expanding population since the Pleistocene, approximately 100,000 and 60,000 years ago for *P*. *capensis* and *P*. *peringueyi* respectively. Both methodologies have limitations, for example with mismatch distribution parameters being susceptible to early branching in the gene tree [4], and BSP plots being limited by the over-interpretation of BSP curves [79]. However, the concordant estimates suggest that both *P*. *capensis* and *P*. *peringueyi* retained demographically stable populations through the LGM, with the frequent and dramatic climatic changes of the Pleistocene having a stronger impact in shaping the biogeographic and demographic history of both species. Similar Pleistocene population expansion estimates have been reported for other South African coastal organisms, including the Cape sea urchin *Parechinus angulosus* [32], the clinid species *Clinus cottoides*, *C*. *superciliosus* and *Muraenoclinus dorsalis* [37], and the southern African barnacle *Tetraclita serrata* [41].

Analysis of genetic diversity metrics across the sampled biogeographic regions indicates that genetic diversity varies across regions, with diversity typically being highest on the south coast in both *P*. *capensis* and *P*. *peringueyi*; a pattern that is typical within other southern African coastal organisms [32], [41]. One explanation for this pattern of higher genetic diversity in the south is that in both species large populations have been maintained on the south coast over evolutionary time, potentially due to the stability of the habitat [80]. Both species have been shown to maintain large contemporary population sizes ([46], Wood pers. obs.), thus if contemporary patterns are reflective of evolutionary history, large population sizes are likely to have contributed to high levels of genetic diversity. Alternatively, this region of higher genetic diversity could also signal a refugium during glacial low sea-level stands. This scenario is supported by the occurrence of the highest number of private haplotypes on the south coast for both species, which is indicative of higher genetic heterozygosity in refugia. In contrast, south-east coast populations in *P*. *capensis* and west coast populations in *P*. *peringueyi* are characterised by only a few common haplotypes, a pattern that reflects a more recent founder event than when compared to the southern biogeographic region.

However, it is important is note that the demographic signals exhibited by *P*. *capensis* and *P*. *peringueyi* do differ to some degree, which is likely a reflection of species specific life history strategies. The star-burst like network of *P*. *capensis* and lower level of haplotypic diversity observed in this species can potentially be explained by environmental tolerance. Whilst *P*. *peringueyi* is a generalist and thus able to survive in greater portions of available habitat during periods of climatic change, *P*. *capensis* is far more specialist and thus far more susceptible to environmental disturbance.

## Supporting information

S1 Fig A bayesian phylogram for the CO1 haplotypes in [50] and CO1 haplotypes herein. Statistical values above the nodes represent the posterior probablity (pP) values for the Bayesian analyses. Values below each node represent the bootstrapping values for maximum likelihood. Only bootstrap values >75% and pP values > 0.95 are shown. An asterisk (*) indicate clades that were not statistically supported.
(TIF)

S1 Table The molecular markers and primer pairs used in this study with their respective polymerase chain reaction conditions. Temperatures in bold under PCR profile indicate the annealing temperatures. The final extension was at 72°C for 10 minutes. * Protein coding.
(DOCX)

S2 Table Haplotype table showing the distribution of haplotypes across sampling localities for *Palaemon capensis*. (DOCX)

S3 Table Haplotype table showing the distribution of haplotypes across sampling localities for *Palaemon peringueyi*. (DOCX)

S4 Table Pairwise ΦST values for the CO1 locus in across all 12 of the different sample localities for *Palaemon capensis*. (DOCX)

S5 Table Pairwise ΦST values for the CO1 locus in across all 24 of the different sample localities for *Palaemon peringueyi*. (DOCX)
