# Supplementary figures and images for: Phylogeographic patterning among two codistributed shrimp species (Crustacea: Decapoda: Palaemonidae) reveals high levels of connectivity across biogeographic regions along the South African coast
Source: PLoS One. 2017 Mar 10;12(3):e0173356. doi: 10.1371/journal.pone.0173356 (PMC5345795; doi:10.1371/journal.pone.0173356)

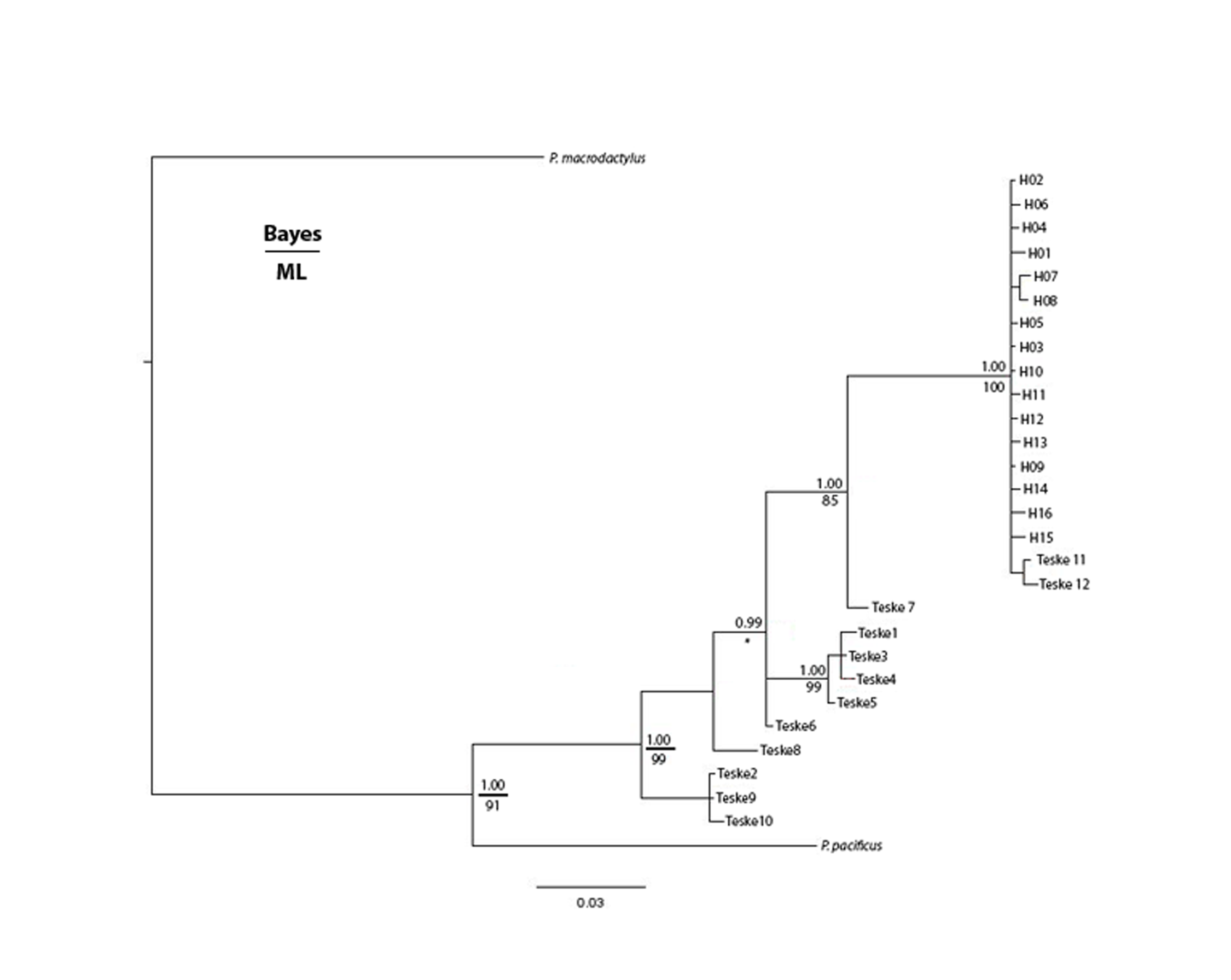

Supplement: S1 Fig — Statistical values above the nodes represent the posterior probablity (pP) values for the Bayesian analyses. Values below each node represent the bootstrapping values for maximum likelihood. Only bootstrap values >75% and pP values > 0.95 are shown. An asterisk (*) indicate clades that were not statistically supported. (TIF) [file pone.0173356.s001.tif]
