# Supplementary material for: Phylogeographic patterning among two codistributed shrimp species (Crustacea: Decapoda: Palaemonidae) reveals high levels of connectivity across biogeographic regions along the South African coast
Source: PLoS One. 2017 Mar 10;12(3):e0173356. doi: 10.1371/journal.pone.0173356 (PMC5345795; doi:10.1371/journal.pone.0173356)
Supplement: S1 Table — Temperatures in bold under PCR profile indicate the annealing temperatures. The final extension was at 72°C for 10 minutes. * Protein coding. (DOCX) [file pone.0173356.s002.docx]

**S1 Table**. The molecular marker and primer pairs used in this study with their respective polymerase chain reaction conditions. Temperatures in bold under PCR profile indicate the annealing temperatures. The final extension was at 72 °C for 10 minutes. * Protein coding.

| Molecular markers | Product size (pb) | PCR | Primer sequence (5’-3’ direction) | PCR profile | Reference |
| --- | --- | --- | --- | --- | --- |
| *Cytochrome oxidase subunit I |  | LCOI-1490  HCOI-2198  Shrimp-F  Shrimp-R | GGTCAACAAATCATAAAGATATTG  TAAACTTCAGGGTGACCAAAAAATCA  CGTCACAGCCCATGCATTC  TAGAGAATCGGGTCTCCTCCT | 94 °C (4 min.), [94 °C (30 sec.), 42 °C (35 sec.), 72 °C (45 sec.)] x 36 | Folmer et al*.* (1994)  Present study |
