# Supplementary material for: Phylogeographic patterning among two codistributed shrimp species (Crustacea: Decapoda: Palaemonidae) reveals high levels of connectivity across biogeographic regions along the South African coast
Source: PLoS One. 2017 Mar 10;12(3):e0173356. doi: 10.1371/journal.pone.0173356 (PMC5345795; doi:10.1371/journal.pone.0173356)
Supplement: S2 Table — (DOCX) [file pone.0173356.s003.docx]

| **Table S2.** Haplotype table showing the distribution of haplotypes across sampling localities for *Palaemon capensis*.   \|  \| **H1** \| **H2** \| **H3** \| **H4** \| **H5** \| **H6** \| **H7** \| **H8** \| **H9** \| **H10** \| **H11** \| **H12** \| **H13** \| **H14** \| **H15** \| **H16** \| **H17** \| **H18** \| **H19** \| **H20** \| **H21** \| **H22** \| \| --- \| --- \| --- \| --- \| --- \| --- \| --- \| --- \| --- \| --- \| --- \| --- \| --- \| --- \| --- \| --- \| --- \| --- \| --- \| --- \| --- \| --- \| --- \| \| Swellendam \|  \|  \| 3 \|  \|  \| 1 \| 2 \|  \|  \|  \|  \|  \|  \| 1 \|  \|  \|  \|  \|  \|  \|  \|  \| \| Voorhuis \| 1 \|  \| 4 \|  \|  \|  \|  \| 1 \|  \|  \| 1 \|  \|  \| 1 \|  \|  \|  \|  \|  \| 1 \|  \|  \| \| Malgas \|  \|  \| 6 \|  \|  \|  \|  \| 1 \|  \|  \|  \|  \|  \| 1 \|  \|  \|  \|  \|  \|  \|  \| 1 \| \| Duiwenhoks \|  \|  \| 4 \|  \|  \|  \| 1 \|  \| 1 \| 1 \|  \|  \|  \| 1 \|  \|  \|  \| 1 \| 1 \|  \|  \|  \| \| Goukou \|  \| 1 \| 4 \|  \|  \|  \|  \| 1 \|  \|  \|  \| 1 \|  \| 2 \|  \|  \|  \| 1 \|  \|  \|  \|  \| \| Gourits \|  \|  \| 5 \|  \|  \| 1 \|  \|  \|  \|  \|  \|  \|  \|  \| 1 \| 1 \|  \|  \|  \|  \|  \|  \| \| Little Brak \|  \|  \| 2 \|  \| 2 \|  \|  \| 1 \| 2 \|  \|  \|  \| 1 \| 1 \|  \|  \| 1 \|  \|  \|  \|  \|  \| \| Knysna \|  \|  \| 1 \|  \|  \|  \|  \|  \|  \|  \|  \|  \|  \|  \|  \|  \|  \|  \|  \|  \|  \|  \| \| Keurbooms \|  \|  \| 7 \|  \|  \|  \|  \|  \|  \|  \|  \|  \|  \|  \|  \|  \|  \|  \|  \|  \| 1 \|  \| \| Sundays \|  \|  \| 10 \|  \|  \|  \|  \|  \|  \|  \|  \|  \|  \|  \|  \|  \|  \|  \|  \|  \|  \|  \| \| Kowie \|  \|  \| 9 \| 1 \|  \|  \|  \|  \|  \|  \|  \|  \|  \|  \|  \|  \|  \|  \|  \|  \|  \|  \| \| Kieskamma \|  \|  \| 9 \|  \|  \|  \|  \|  \|  \|  \|  \|  \|  \|  \|  \|  \|  \|  \|  \|  \|  \|  \| |  |  |
| --- | --- | --- | --- | --- | --- | --- | --- | --- | --- | --- | --- | --- | --- | --- | --- | --- | --- | --- | --- | --- | --- | --- | --- | --- | --- | --- | --- | --- | --- | --- | --- | --- | --- | --- | --- | --- | --- | --- | --- | --- | --- | --- | --- | --- | --- | --- | --- | --- | --- | --- | --- | --- | --- | --- | --- | --- | --- | --- | --- | --- | --- | --- | --- | --- | --- | --- | --- | --- | --- | --- | --- | --- | --- | --- | --- | --- | --- | --- | --- | --- | --- | --- | --- | --- | --- | --- | --- | --- | --- | --- | --- | --- | --- | --- | --- | --- | --- | --- | --- | --- | --- | --- | --- | --- | --- | --- | --- | --- | --- | --- | --- | --- | --- | --- | --- | --- | --- | --- | --- | --- | --- | --- | --- | --- | --- | --- | --- | --- | --- | --- | --- | --- | --- | --- | --- | --- | --- | --- | --- | --- | --- | --- | --- | --- | --- | --- | --- | --- | --- | --- | --- | --- | --- | --- | --- | --- | --- | --- | --- | --- | --- | --- | --- | --- | --- | --- | --- | --- | --- | --- | --- | --- | --- | --- | --- | --- | --- | --- | --- | --- | --- | --- | --- | --- | --- | --- | --- | --- | --- | --- | --- | --- | --- | --- | --- | --- | --- | --- | --- | --- | --- | --- | --- | --- | --- | --- | --- | --- | --- | --- | --- | --- | --- | --- | --- | --- | --- | --- | --- | --- | --- | --- | --- | --- | --- | --- | --- | --- | --- | --- | --- | --- | --- | --- | --- | --- | --- | --- | --- | --- | --- | --- | --- | --- | --- | --- | --- | --- | --- | --- | --- | --- | --- | --- | --- | --- | --- | --- | --- | --- | --- | --- | --- | --- | --- | --- | --- | --- | --- | --- | --- | --- | --- | --- | --- | --- | --- | --- | --- | --- | --- | --- | --- | --- | --- | --- | --- | --- | --- | --- | --- | --- | --- | --- | --- | --- | --- | --- | --- | --- | --- |
