# Supplementary material for: Phylogeographic patterning among two codistributed shrimp species (Crustacea: Decapoda: Palaemonidae) reveals high levels of connectivity across biogeographic regions along the South African coast
Source: PLoS One. 2017 Mar 10;12(3):e0173356. doi: 10.1371/journal.pone.0173356 (PMC5345795; doi:10.1371/journal.pone.0173356)
Supplement: S3 Table — (DOCX) [file pone.0173356.s004.docx]

| **Table S3.** Haplotype table showing the distribution of haplotypes across sampling localities for *Palaemon peringueyi*.   \|  \| **H1** \| **H2** \| **H3** \| **H4** \| **H5** \| **H6** \| **H7** \| **H8** \| **H9** \| **H10** \| **H11** \| **H12** \| **H13** \| **H14** \| **H15** \| **H16** \| \| --- \| --- \| --- \| --- \| --- \| --- \| --- \| --- \| --- \| --- \| --- \| --- \| --- \| --- \| --- \| --- \| --- \| \| Olifants \|  \| 3 \| 3 \|  \|  \| 1 \| 1 \|  \| 2 \|  \|  \|  \|  \|  \|  \|  \| \| Berg \|  \| 5 \| 4 \|  \|  \|  \| 2 \|  \| 1 \|  \|  \|  \|  \|  \|  \|  \| \| Langebaan \|  \| 2 \| 8 \|  \|  \|  \|  \|  \|  \|  \|  \|  \|  \|  \|  \|  \| \| Rooiels \|  \| 3 \| 3 \|  \|  \|  \|  \|  \| 2 \| 2 \|  \|  \|  \|  \|  \|  \| \| Palmiet \|  \| 2 \| 2 \|  \|  \|  \|  \|  \| 4 \| 2 \|  \|  \|  \|  \|  \|  \| \| Goukou \|  \| 4 \| 2 \|  \|  \|  \|  \|  \| 2 \|  \|  \|  \|  \|  \|  \|  \| \| Great Brak \|  \| 1 \| 4 \| 1 \|  \|  \|  \|  \| 1 \| 3 \|  \|  \|  \|  \|  \|  \| \| Touw \|  \| 3 \| 4 \|  \|  \|  \|  \|  \|  \| 1 \|  \| 1 \|  \|  \|  \|  \| \| Swartvlei \|  \| 2 \| 1 \|  \|  \|  \|  \|  \| 5 \| 1 \| 1 \|  \|  \|  \|  \|  \| \| Knysna \|  \| 1 \| 3 \|  \| 1 \|  \|  \|  \| 3 \| 2 \|  \|  \|  \|  \|  \|  \| \| Goukamma \|  \| 3 \| 1 \|  \|  \|  \| 3 \|  \| 1 \|  \|  \|  \|  \|  \|  \|  \| \| Gamtoos \|  \| 2 \| 3 \|  \|  \|  \|  \|  \| 4 \| 1 \|  \|  \|  \|  \|  \|  \| \| Sundays \|  \| 3 \| 4 \|  \|  \|  \|  \|  \|  \| 1 \|  \| 1 \|  \|  \|  \|  \| \| Bushmans \|  \|  \| 4 \|  \|  \|  \|  \|  \| 6 \|  \|  \|  \|  \|  \|  \|  \| \| Riet \| 1 \|  \| 3 \|  \| 1 \|  \| 1 \|  \| 1 \|  \|  \|  \|  \|  \|  \|  \| \| East Kleinmond \|  \| 2 \| 2 \|  \|  \|  \|  \|  \| 2 \| 1 \|  \|  \|  \|  \|  \|  \| \| Fish \|  \| 1 \| 3 \|  \|  \|  \|  \|  \| 1 \| 1 \|  \| 2 \| 1 \|  \|  \|  \| \| Old Woman’s \|  \| 2 \| 7 \|  \|  \|  \|  \|  \|  \| 1 \|  \|  \|  \|  \|  \|  \| \| Nahoon \|  \| 1 \| 3 \|  \|  \|  \|  \|  \| 1 \| 2 \|  \| 2 \|  \|  \|  \|  \| \| Chula \|  \|  \|  \|  \|  \|  \| 1 \|  \| 4 \| 2 \|  \|  \|  \|  \|  \|  \| \| Kei \|  \| 2 \| 2 \|  \| 1 \|  \|  \|  \| 1 \| 4 \|  \|  \|  \|  \|  \|  \| \| Qwaninga \|  \|  \| 2 \|  \|  \|  \| 2 \| 1 \| 2 \|  \|  \|  \|  \|  \|  \|  \| \| Mtata \|  \|  \|  \|  \|  \|  \|  \|  \| 5 \|  \|  \|  \|  \| 2 \|  \|  \| \| Umganzana \|  \|  \|  \|  \|  \|  \|  \|  \| 4 \|  \|  \|  \|  \| 2 \| 1 \| 1 \| |  |  |
| --- | --- | --- | --- | --- | --- | --- | --- | --- | --- | --- | --- | --- | --- | --- | --- | --- | --- | --- | --- | --- | --- | --- | --- | --- | --- | --- | --- | --- | --- | --- | --- | --- | --- | --- | --- | --- | --- | --- | --- | --- | --- | --- | --- | --- | --- | --- | --- | --- | --- | --- | --- | --- | --- | --- | --- | --- | --- | --- | --- | --- | --- | --- | --- | --- | --- | --- | --- | --- | --- | --- | --- | --- | --- | --- | --- | --- | --- | --- | --- | --- | --- | --- | --- | --- | --- | --- | --- | --- | --- | --- | --- | --- | --- | --- | --- | --- | --- | --- | --- | --- | --- | --- | --- | --- | --- | --- | --- | --- | --- | --- | --- | --- | --- | --- | --- | --- | --- | --- | --- | --- | --- | --- | --- | --- | --- | --- | --- | --- | --- | --- | --- | --- | --- | --- | --- | --- | --- | --- | --- | --- | --- | --- | --- | --- | --- | --- | --- | --- | --- | --- | --- | --- | --- | --- | --- | --- | --- | --- | --- | --- | --- | --- | --- | --- | --- | --- | --- | --- | --- | --- | --- | --- | --- | --- | --- | --- | --- | --- | --- | --- | --- | --- | --- | --- | --- | --- | --- | --- | --- | --- | --- | --- | --- | --- | --- | --- | --- | --- | --- | --- | --- | --- | --- | --- | --- | --- | --- | --- | --- | --- | --- | --- | --- | --- | --- | --- | --- | --- | --- | --- | --- | --- | --- | --- | --- | --- | --- | --- | --- | --- | --- | --- | --- | --- | --- | --- | --- | --- | --- | --- | --- | --- | --- | --- | --- | --- | --- | --- | --- | --- | --- | --- | --- | --- | --- | --- | --- | --- | --- | --- | --- | --- | --- | --- | --- | --- | --- | --- | --- | --- | --- | --- | --- | --- | --- | --- | --- | --- | --- | --- | --- | --- | --- | --- | --- | --- | --- | --- | --- | --- | --- | --- | --- | --- | --- | --- | --- | --- | --- | --- | --- | --- | --- | --- | --- | --- | --- | --- | --- | --- | --- | --- | --- | --- | --- | --- | --- | --- | --- | --- | --- | --- | --- | --- | --- | --- | --- | --- | --- | --- | --- | --- | --- | --- | --- | --- | --- | --- | --- | --- | --- | --- | --- | --- | --- | --- | --- | --- | --- | --- | --- | --- | --- | --- | --- | --- | --- | --- | --- | --- | --- | --- | --- | --- | --- | --- | --- | --- | --- | --- | --- | --- | --- | --- | --- | --- | --- | --- | --- | --- | --- | --- | --- | --- | --- | --- | --- | --- | --- | --- | --- | --- | --- | --- | --- | --- | --- | --- | --- | --- | --- | --- | --- | --- | --- | --- | --- | --- | --- | --- | --- | --- | --- | --- | --- | --- | --- | --- | --- | --- | --- | --- | --- | --- | --- | --- | --- |
