# Supplementary material for: Phylogeographic patterning among two codistributed shrimp species (Crustacea: Decapoda: Palaemonidae) reveals high levels of connectivity across biogeographic regions along the South African coast
Source: PLoS One. 2017 Mar 10;12(3):e0173356. doi: 10.1371/journal.pone.0173356 (PMC5345795; doi:10.1371/journal.pone.0173356)
Supplement: S4 Table — (DOCX) [file pone.0173356.s005.docx]

**Table S4. Pairwise Φ_ST_ values for the CO1 locus in across all 12 of the different sample localities for *Palaemon capensis*.**

|  | 1 | 2 | 3 | 4 | 5 | 6 | 7 | 8 | 9 | 10 | 11 | 12 |
| --- | --- | --- | --- | --- | --- | --- | --- | --- | --- | --- | --- | --- |
| 1 | - |  |  |  |  |  |  |  |  |  |  |  |
| 2 | -0.029 | - |  |  |  |  |  |  |  |  |  |  |
| 3 | -0.029 | -0.063 | - |  |  |  |  |  |  |  |  |  |
| 4 | -0.070 | -0.039 | -0.042 | - |  |  |  |  |  |  |  |  |
| 5 | -0.028 | -0.070 | -0.060 | -0.062 | - |  |  |  |  |  |  |  |
| 6 | 0.059 | -0.017 | -0.016 | 0.048 | 0.021 | - |  |  |  |  |  |  |
| 7 | 0.0210 | -0.012 | -0.014 | 0.006 | 0.006 | -0.025 | - |  |  |  |  |  |
| 8 | -0.599 | -0.779 | -0.749 | -0.637 | -0.601 | -0.886 | -0.886 | - |  |  |  |  |
| 9 | 0.004 | -0.028 | -0.029 | 0.008 | 0.011 | -0.028 | -0.004 | -1.000 | - |  |  |  |
| 10 | **0.270** | **0.126** | 0.142 | 0.181 | 0.200 | 0.091 | **0.099** | 0.000 | 0.029 | - |  |  |
| 11 | **0.222** | **0.113** | 0.125 | 0.166 | 0.182 | 0.072 | 0.091 | -1.000 | 0.017 | 0.000 | - |  |
| 12 | **0.249** | 0.110 | 0.125 | 0.165 | 0.183 | 0.075 | 0.085 | 0.000 | 0.016 | 0.000 | -0.011 |  |

Statistically significant values (*p* < 0.05) are highlighted in bold.

1 = Swellendam; 2 = Voorhuis; 3 = Malgas; 4 = Duiwenhoks; 5 = Goukou; 6 = Gourits; 7 = Little Brak; 8 = Knysna; 9 = Keurbooms; 10 = Sundays; 11 = Kowie; 12 = Kieskamma
