# Supplementary material for: Phylogeographic patterning among two codistributed shrimp species (Crustacea: Decapoda: Palaemonidae) reveals high levels of connectivity across biogeographic regions along the South African coast
Source: PLoS One. 2017 Mar 10;12(3):e0173356. doi: 10.1371/journal.pone.0173356 (PMC5345795; doi:10.1371/journal.pone.0173356)
Supplement: S5 Table — (DOCX) [file pone.0173356.s006.docx]

**Table S5. Pairwise Φ_ST_ values for the CO1 locus in across all 24 of the different sample localities for *Palaemon peringueyi***.

|  | 13 | 14 | 15 | 16 | 17 | 18 | 19 | 20 | 21 | 22 | 23 | 24 | 25 | 26 | 27 | 28 | 29 | 30 | 31 | 32 | 33 | 34 | 35 | 36 |  |
| --- | --- | --- | --- | --- | --- | --- | --- | --- | --- | --- | --- | --- | --- | --- | --- | --- | --- | --- | --- | --- | --- | --- | --- | --- | --- |
| 13 |  |  |  |  |  |  |  |  |  |  |  |  |  |  |  |  |  |  |  |  |  |  |  |  | |
| 14 | -0.067 |  |  |  |  |  |  |  |  |  |  |  |  |  |  |  |  |  |  |  |  |  |  |  | |
| 15 | 0.067 | 0.128 |  |  |  |  |  |  |  |  |  |  |  |  |  |  |  |  |  |  |  |  |  |  | |
| 16 | -0.018 | 0.057 | 0.017 |  |  |  |  |  |  |  |  |  |  |  |  |  |  |  |  |  |  |  |  |  | |
| 17 | 0.043 | 0.151 | **0.130** | -0.062 |  |  |  |  |  |  |  |  |  |  |  |  |  |  |  |  |  |  |  |  | |
| 18 | -0.100 | -0.029 | **0.112** | -0.039 | 0.021 |  |  |  |  |  |  |  |  |  |  |  |  |  |  |  |  |  |  |  | |
| 19 | 0.053 | 0.134 | 0.065 | -0.091 | -0.062 | 0.051 |  |  |  |  |  |  |  |  |  |  |  |  |  |  |  |  |  |  | |
| 20 | 0.015 | 0.053 | 0.006 | -0.062 | 0.053 | 0.025 | -0.041 |  |  |  |  |  |  |  |  |  |  |  |  |  |  |  |  |  | |
| 21 | 0.070 | 0.179 | **0.186** | -0.017 | -0.093 | 0.046 | -0.017 | 0.106 |  |  |  |  |  |  |  |  |  |  |  |  |  |  |  |  | |
| 22 | 0.054 | 0.146 | 0.106 | -0.082 | -0.092 | 0.043 | -0.101 | -0.001 | -0.060 |  |  |  |  |  |  |  |  |  |  |  |  |  |  |  | |
| 23 | 0.029 | -0.026 | **0.337** | 0.206 | **0.268** | 0.120 | **0.267** | **0.207** | **0.273** | **0.265** |  |  |  |  |  |  |  |  |  |  |  |  |  |  | |
| 24 | 0.030 | 0.144 | **0.119** | -0.049 | -0.101 | 0.004 | -0.030 | 0.075 | -0.083 | -0.062 | **0.272** |  |  |  |  |  |  |  |  |  |  |  |  |  | |
| 25 | 0.015 | 0.053 | 0.006 | -0.062 | 0.053 | 0.025 | -0.041 | -0.125 | 0.106 | 0.001 | **0.207** | 0.075 |  |  |  |  |  |  |  |  |  |  |  |  | |
| 26 | 0.249 | **0.376** | **0.445** | 0.193 | 0.034 | **0.282** | **0.193** | **0.357** | -0.000 | 0.124 | **0.471** | 0.011 | **0.357** |  |  |  |  |  |  |  |  |  |  |  | |
| 27 | -0.082 | -0.063 | 0.032 | -0.060 | 0.001 | -0.064 | -0.022 | -0.049 | 0.031 | -0.013 | 0.002 | 0.007 | -0.049 | **0.219** |  |  |  |  |  |  |  |  |  |  | |
| 28 | -0.050 | 0.046 | 0.032 | -0.127 | -0.114 | -0.079 | -0.097 | -0.041 | -0.079 | -0.106 | 0.184 | -0.114 | -0.041 | 0.111 | -0.079 |  |  |  |  |  |  |  |  |  | |
| 29 | 0.155 | 0.219 | 0.159 | 0.014 | 0.043 | **0.171** | -0.035 | -0.011 | 0.077 | -0.018 | **0.310** | 0.089 | -0.011 | **0.278** | 0.048 | 0.020 |  |  |  |  |  |  |  |  | |
| 30 | 0.061 | 0.119 | -0.085 | -0.032 | 0.082 | 0.094 | -0.008 | -0.053 | 0.143 | 0.039 | **0.312** | 0.087 | -0.053 | **0.395** | 0.004 | -0.012 | 0.078 |  |  |  |  |  |  |  | |
| 31 | 0.163 | 0.229 | 0.175 | 0.014 | 0.045 | **0.186** | -0.040 | -0.012 | 0.082 | -0.021 | **0.327** | 0.095 | -0.012 | **0.299** | 0.053 | 0.025 | -0.115 | 0.085 |  |  |  |  |  |  | |
| 32 | 0.086 | **0.185** | **0.254** | 0.021 | -0.073 | 0.103 | 0.003 | 0.139 | -0.088 | -0.047 | 0.220 | 0.048 | 0.139 | -0.011 | 0.013 | -0.041 | 0.065 | 0.199 | 0.071 |  |  |  |  |  | |
| 33 | 0.124 | 0.176 | **0.195** | -0.017 | 0.051 | 0.142 | -0.053 | -0.020 | 0.092 | -0.029 | **0.282** | 0.111 | -0.020 | **0.364** | 0.016 | 0.011 | -0.035 | 0.093 | 0.039 | -0.092 |  |  |  |  | |
| 34 | -0.022 | -0.014 | **0.192** | 0.052 | 0.070 | 0.042 | 0.082 | 0.090 | 0.078 | 0.072 | -0.059 | 0.079 | 0.090 | **0.246** | -0.079 | 0.015 | 0.135 | 0.160 | 0.145 | -0.010 | 0.124 |  |  |  | |
| 35 | **0.442** | **0.544** | **0.727** | **0.453** | **0.304** | **0.511** | 0.453 | **0.578** | **0.215** | **0.378** | **0.570** | **0.316** | **0.578** | **0.261** | **0.396** | **0.401** | **0.471** | **0.671** | **0.502** | 0.167 | **0.548** | **0.376** |  |  | |
| 36 | **0.346** | **0.447** | **0.526** | **0.336** | **0.218** | **0.365** | **0.336** | **0.441** | **0.163** | **0.279** | **0.471** | **0.220** | **0.441** | **0.167** | **0.302** | **0.264** | **0.352** | **0.494** | **0.386** | 0.122 | **0.433** | **0.294** | -0.034 |  | |

Statistically significant values (*p* <0.05) are highlighted in bold.

13 = Olifants; 14 = Berg; 15 = Langebaan; 16 = Rooiels; 17 =Palmiet; 18 = Goukou; 19 = Great Brak; 20 = Touw; 21 = Swartvlei; 22 = Knysna; 23 = Goukamma; 24 = Gamtoos; 25 = Sundays; 26 = Bushmans; 27 = Riet; 28 = East Kleinmond; 29 = Fish; 30 = Old Woman’s; 31 = Nahoon; 32 = Chula; 33 = Kei; 34 = Qwaninga; 35 = Mtata; 36 = Umganzana
